# Comparison and evaluation of two exome capture kits and sequencing platforms for variant calling

**DOI:** 10.1186/s12864-015-1796-6

**Published:** 2015-08-05

**Authors:** Guoqiang Zhang, Jianfeng Wang, Jin Yang, Wenjie Li, Yutian Deng, Jing Li, Jun Huang, Songnian Hu, Bing Zhang

**Affiliations:** Core Genomic Facility and CAS Key Laboratory of Genome Sciences & Information, Beijing Institute of Genomics, Chinese Academy of Sciences, Beijing, 100101 China; CAS Key Laboratory of Genome Sciences & Information, Beijing Institute of Genomics, Chinese Academy of Sciences, Beijing, 100101 China

**Keywords:** Exome sequencing, Variant calling, Accuracy

## Abstract

**Background:**

To promote the clinical application of next-generation sequencing, it is important to obtain accurate and consistent variants of target genomic regions at low cost. Ion Proton, the latest updated semiconductor-based sequencing instrument from Life Technologies, is designed to provide investigators with an inexpensive platform for human whole exome sequencing that achieves a rapid turnaround time. However, few studies have comprehensively compared and evaluated the accuracy of variant calling between Ion Proton and Illumina sequencing platforms such as HiSeq 2000, which is the most popular sequencing platform for the human genome. The Ion Proton sequencer combined with the Ion TargetSeq™ Exome Enrichment Kit together make up TargetSeq-Proton, whereas SureSelect-Hiseq is based on the Agilent SureSelect Human All Exon v4 Kit and the HiSeq 2000 sequencer.

**Results:**

Here, we sequenced exonic DNA from four human blood samples using both TargetSeq-Proton and SureSelect-HiSeq. We then called variants in the exonic regions that overlapped between the two exome capture kits (33.6 Mb). The rates of shared variant loci called by two sequencing platforms were from 68.0 to 75.3 % in four samples, whereas the concordance of co-detected variant loci reached 99 %. Sanger sequencing validation revealed that the validated rate of concordant single nucleotide polymorphisms (SNPs) (91.5 %) was higher than the SNPs specific to TargetSeq-Proton (60.0 %) or specific to SureSelect-HiSeq (88.3 %). With regard to 1-bp small insertions and deletions (InDels), the Sanger sequencing validated rates of concordant variants (100.0 %) and SureSelect-HiSeq-specific (89.6 %) were higher than those of TargetSeq-Proton-specific (15.8 %).

**Conclusions:**

In the sequencing of exonic regions, a combination of using of two sequencing strategies (SureSelect-HiSeq and TargetSeq-Proton) increased the variant calling specificity for concordant variant loci and the sensitivity for variant loci called by any one platform. However, for the sequencing of platform-specific variants, the accuracy of variant calling by HiSeq 2000 was higher than that of Ion Proton, specifically for the InDel detection. Moreover, the variant calling software also influences the detection of SNPs and, specifically, InDels in Ion Proton exome sequencing.

**Electronic supplementary material:**

The online version of this article (doi:10.1186/s12864-015-1796-6) contains supplementary material, which is available to authorized users.

## Background

Whole genome sequencing (WGS) comprehensively investigates genome sequence changes such as single-nucleotide variants (SNVs) [1, 2], insertions and deletions (InDels) [3–9], chromosomal rearrangements [10, 11], and copy-number variation [12, 13], and so on. However, whole exome sequencing (WES) has become more popular because exons are more interpretable than other genomic regions and because the technique allows more samples to be analysed. Previous studies have analysed disease loci that segregate in families [14–16], and large disease cohorts (e.g., National Heart, Lung, and Blood Institute), and validated their findings in WGS studies [16] using exome sequencing technology. Other groups have assessed the clinical application of next generation sequencing (NGS) by target sequencing [17–19].

With recent advances in NGS technology [2, 3, 6, 17–19], it is now possible to sequence the whole genomic or exonic DNA of an individual. Compared with traditional single nucleotide polymorphism (SNP) arrays [20], WGS can generate target DNA sequences and identify substantially more genetic variations, thus explaining a larger fraction of human phenotypic diversity [21].

Currently, the most widely used sequencing platform in human genome sequencing research is the Illumina HiSeq series of instruments (HiSeq 2000/2500), which use highly-parallel optical sensing of polymerization reactions to achieve an ultra-high throughput (up to 6000 million reads per run with paired-end sequencing). Life Technologies has also released a new version of the semiconductor sequencing platform, Ion Proton (Proton), which provides researchers with an alternative sequencing platform. Proton has a medium-throughput, cost effectiveness and rapid turnaround time (just 4 h of sequencing run time). Thus, Proton is an attractive means of validating the variants called in whole genomes by other sequencing platforms [22], sequencing of whole exomes [23], screening cancer-related genes in solid tumours [24], or conducting sequencing-based clinical applications such as prenatal diagnosis which has a strict turn-around time requirement [25]. However, given the differences between sequencing technologies and subsequent variant calling pipelines applied by HiSeq 2000 and Proton, it is necessary to comprehensively compare the two platforms.

Previously, the variant calling performance of the Proton sequencer was assessed by comparing it with variants called by HiSeq 2000 [23], Complete Genomics, and Illumina SNP microarray. Another team used the Proton sequencer to validate the whole exome variants called by WGS on the HiSeq 2500 sequencer [22]. In the present study, we comprehensively compared the differences between variants called by HiSeq 2000 with the Agilent SureSelect Human All Exon v4 kit (SureSelect-HiSeq) and Proton with the Ion TargetSeq™ Exome Enrichment kit (TargetSeq-Proton), and validated the variants by Sanger sequencing. Our results show that there is a significant discrepancy between SureSelect-HiSeq and TargetSeq-Proton sequencing strategies, and provide some guidance for analysing personal genome on different sequencing platforms.

## Results

### Data summary of exome sequencing

Exonic DNA from four individual Chinese genomic DNA samples was captured by the Ion TargetSeq™ Exome Enrichment Kit using probes of various lengths (85.1 ± 64.1), and subsequently sequenced by the Proton sequencing platform (Life Technologies). We obtained approximately 40× average sequence coverage on targeted regions (Table 1). For all samples, Proton reads covered more than 90 % of the targeted region with ≥10× reads coverage.

**Table 1:** Statistics of reads and alignment to reference genome for four samples’s exome sequencing on TargetSeq-Proton/SureSelect-HiSeq platform

Sample	Sequencing platform	Total reads (M)	Total bases (Gb)	Total mapped reads (M)	Average read length (bp)	Average coverage depth	Coverage at 1× (%)	Coverage at 5× (%)	Coverage at 10× (%)	Coverage at 20× (%)
S1	TargetSeq-Proton	48.2	4.5	42.9	94.1	39.8	97	94	90	79
	SureSelect-HiSeq	78.3	7.8	77.4	2*100	40.0	100	97	90	71
S2	TargetSeq-Proton	62.4	6.1	55.1	97.4	51.9	98	95	93	86
	SureSelect-HiSeq	79.9	7.9	79.1	2*100	45.3	100	98	91	74
S3	TargetSeq-Proton	61.3	5.5	55.3	90.9	50.3	97	94	91	84
	SureSelect-HiSeq	56.8	5.6	56.2	2*100	33.4	100	96	86	63
S4	TargetSeq-Proton	53.9	5.2	49.4	96.9	49.8	97	94	91	84
	SureSelect-HiSeq	59.5	5.9	58.9	2*100	36.0	100	96	87	66

Exonic DNA from the same four samples was separately enriched by SureSelect Human All Exon V4 with 120 bp probes, then sequenced on HiSeq 2000 with 2*100 bp read lengths. We obtained ≥33× sequence coverage on targeted regions (Table 1, Additional file 1: Figure S1), and more than 86 % of target regions had ≥10× reads coverage.

### Definition of the evaluation region

We chose the overlapping 33.6 Mb exonic regions as an evaluation region between the Ion TargetSeq™ Exome Enrichment Kit and the Agilent SureSelect V4 Kit. A total of 25,446, 25,413, 25,429, and 25,080 variant loci were detected by Proton and HiSeq 2000 in samples S1, S2, S3, and S4, respectively (Table 2). The co-detected rates of total variant loci were 68.0 %, 75.3 %, 71.7 % and 71.5 %, respectively, on two sequencing platforms for four samples (Table 2). The analyses of the four samples were consistent when evaluating the numbers and co-detection rates of loci observed. Therefore, we randomly chose to describe the results of sample S3 in this report.

**Table 2:** Variant loci detected by TargetSeq-Proton and SureSelect-HiSeq sequencing

	Total loci^a^	Co-detected loci(%)^b^	Concordant loci^c^	Disconcordant loci(TargetSeq-Proton/SureSelect-HiSeq)^d^			
				Hom/Hom	Hom/Het	Het/Hom	Het/Het
S1	25466	17314(68.0)	17202	1	15	93	3
S2	25413	19148(75.3)	19039	2	20	84	3
S3	25429	18222(71.7)	18087	1	26	104	4
S4	25080	17937(71.5)	17808	1	16	111	1

^a^Total loci: all variant loci in the overlapping regions detected by HiSeq 2000 or Ion Proton sequencing, which include the Concordant, Disconcordant, TargetSeq-HiSeq-specific and SureSelect-Proton-specific loci

^b^Co-detected loci: the variant loci co-detected by TargetSeq-HiSeq and SureSelect-Proton sequencing, which include Concordant and Disconcordant loci. The number in parentheses is percentage

^c^Concordant loci: the variant loci with the same genotype detected by between TargetSeq-HiSeq and SureSelect- Proton sequencing

^d^Disconcordant loci: the loci with different variant genotype detected by between TargetSeq-Proton and SureSelect-HiSeq. Hom/Het refers to the loci whose variant genotype is homozygotes detected by TargetSeq-Proton, but heterozygotes detected by SureSelect-HiSeq. Hom/Hom, Het/Hom and Het/Het refer to analogous variant genotype

When considering SNP loci, we evaluated the ratio of transitions to transversions (Ti/Tv) because unusually high or low ratios may be indicative of false positive variants. Overall, Ti/Tv was 2.70 in the total detected SNPs of sample S3 (Additional file 2: Table S1). For concordant SNPs, the Ti/Tv ratio was 3.05. By contrast, notable differences were observed in the ratios of HiSeq 2000-specific (2.02) or Proton-specific (1.93) SNPs, regardless of whether they were novel or known in dbSNP (build 137).

### Comparison of variants-detecting platforms

For sample S3, a total of 25,429 variant loci were detected by Proton or HiSeq 2000, of which 18,222 loci were also detected by Proton and HiSeq 2000 concurrently (Table 2). The concordance of 18,222 co-detected variant loci reached 99.3 % (18,087). Concordance was determined by the loci with the same variant genotype. Among the 18,087 concordant variants, 17,720 SNPs and 367 small InDels were identified. Of the SNPs, 94.9 % (16,810) were reported in dbSNP, while 92.4 % (339) of the small InDels were also reported in this database (Table 3). Of the 5689 total variants only detected by HiSeq 2000, 95.4 % of 4897 SNPs and 90.8 % of 792 small InDels were reported in dbSNP. However, this was true of only 80.2 % of the 1305 SNPs and 12.7 % of the 213 small InDels among the 1518 total variants specific to Proton.

**Table 3:** Pairwise comparison of variants called for four samples by TargetSeq-Proton and SureSelect-HiSeq

	TargetSeq-Proton-specific(dbSNP|novel)^a^			Concordant(dbSNP|novel)			SureSelect-HiSeq-specific(dbSNP|novel)		
	Total	SNPs	InDels	Total	SNPs	InDels	Total	SNPs	InDels
S1	1470 (1021|449)	1274 (998|276)	196 (23|173)	17202 (16288|914)	16833 (15943|890)	369 (345|24)	6682 (6348|334)	5851 (5606|245)	831 (742|89)
S2	1432 (1018|414)	1229 (996|233)	203 (22|181)	19039 (18038|1001)	18655 (17683|972)	384 (355|29)	4833 (4533|300)	4044 (3839|205)	789 (694|95)
S3	1518 (1073|445)	1305 (1046|259)	213 (27|186)	18087 (17149|938)	17720 (16810|910)	367 (339|28)	5689 (5390|299)	4897 (4671|226)	792 (719|73)
S4	1462 (1069|393)	1326 (1051|275)	136 (18|118)	17808 (16891|917)	17409 (16521|888)	399 (370|29)	5681 (5353|328)	4922 (4674|248)	759 (679|80)

^a^The numbers of parentheses refer to known or unknown variant loci in dbSNP databases

**Fig. 1:**
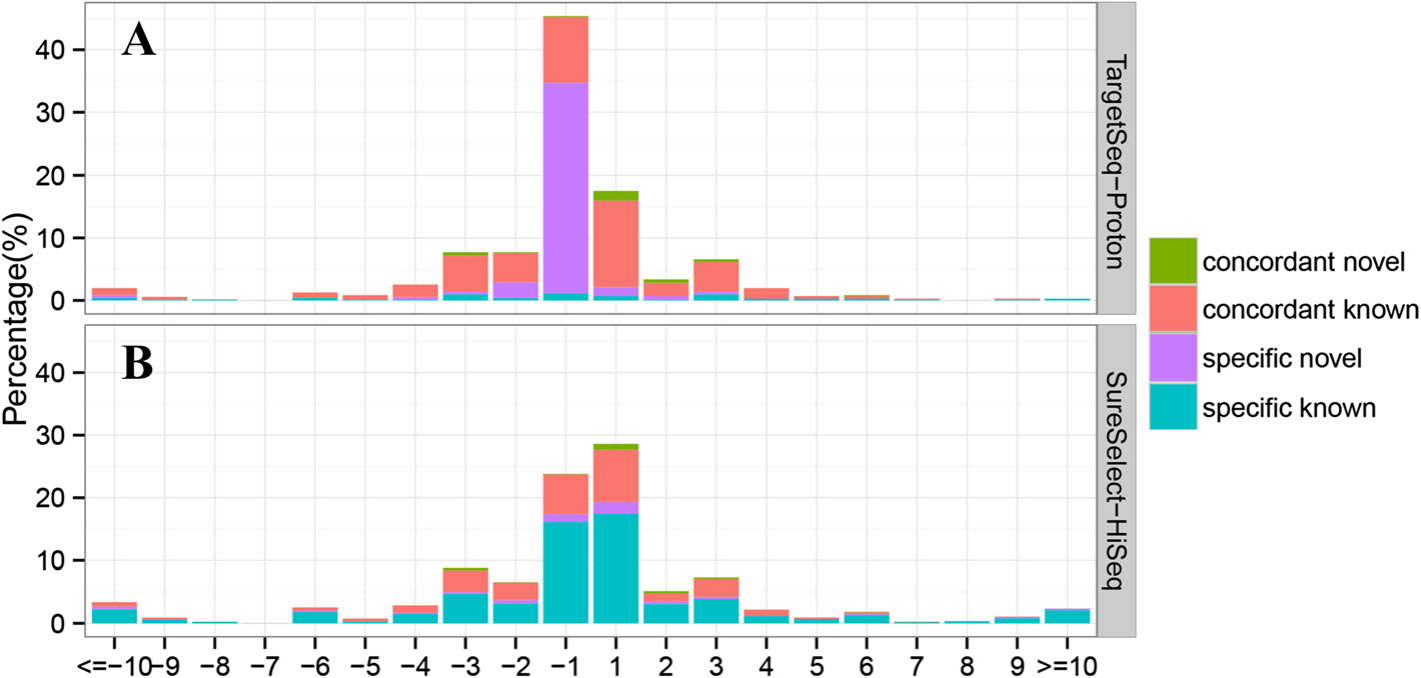
Distribution of size and classification of small InDel called by exome sequencing on SureSelect-HiSeq and TargetSeq-Proton in sample S3. Four classes of small InDel were defined as concordant novel, concordant known, specific novel and specific known. Novel refers to InDels not reported in dbSNP build 137. Known refers to InDels previously reported in dbSNP build 137. **a** showed the size and classification of small InDels called by TargetSeq-Proton. **b** showed the size and classification of small InDels called by SureSelect-HiSeq

We observed a notable difference in the size distribution of InDels calling by the two sequencing platforms as well as the percentage that had been previously reported in dbSNP. Figure 1 shows the size distributions of both concordant and platform-specific InDels. Among all concordant small Indels, 49.0 % (180/367) were 1-bp, which is similar to that of HiSeq 2000-specific (55.3 %). However, this value was 78.4 % for Proton-specific small InDels. Analysis of the composition and homopolymer size of 1-bp InDel loci flanking sequences showed that 1-bp InDels called by Proton were biased toward homopolymer types G and C (Additional file 3: Figure S2).

Among the discordant variant loci, the majority (*n* = 104) were heterozygote calls by Proton but homozygote calls by HiSeq 2000 (Table 2). Additionally, 26 discordant loci were homozygous calls by Proton to heterozygous calls by HiSeq 2000. A few discordant loci consisted of different heterozygotes (*n* = 4) and different homozygotes (*n* = 1) co-detected by Proton and HiSeq 2000.

### Validation by Sanger sequencing

To validate variants called by the two sequencing platforms, we PCR-amplified genomic DNA fragments containing selected SNPs and small InDels, then sequenced them. A total of 240 SNPs of all four samples were randomly selected for validation: 80 HiSeq 2000-specific, 80 Proton-specific and 80 concordant SNPs. Of all 240 SNPs, 69.2 % were successfully amplified and sequenced. The validation rate was 91.5 % for concordant SNPs, 88.3 % for HiSeq 2000-specific and 60.0 % for Proton-specific SNPs (Table 4).

**Table 4:** Sanger sequencing validation comparison on variant subsets of TargetSeq-Proton and SureSelect-HiSeq data calls

	SureSelect-HiSeq-specific		TargetSeq-Proton-specific		Concordant	
	1-bp InDels	SNPs	1-bp InDels	SNPs	1-bp InDels	SNPs
Validated true	89.6 %(60)	88.3 %(53)	15.8 %(6)	60.0 %(21)	100.0 %(47)	91.5 %(65)
Validated false	10.4 %(7)	11.7 %(7)	84.2 %(32)	40.0 %(14)	0.0 %(0)	8.2 %(6)

A total of 240 SNPs and 240 1-bp InDels from four samples were randomly selected for Sanger sequencing validation, with 80 loci from the set of TargetSeq-Proton-specific, 80 from the set of SureSelect-HiSeq-specific, and 80 from the set of concordance between two platforms

As the small InDels biased toward 1-bp InDels (Fig. 1), we selected 80 concordant, 80 HiSeq 2000-specific, and 80 Proton-specific 1-bp InDels for Sanger sequencing validation. Of all 240 InDels, 63.3 % (*n* = 152) were successfully amplified and sequenced. Concordant and HiSeq 2000-specific InDels had validation rates of 100.0 % (*n* = 47) and 89.6 % (*n* = 60), respectively (Table 4). However, the validation rate of 38 Proton-specific InDels was only 15.8 % (*n* = 6).

### Comparisons of variants-detecting pipelines

For the Proton sequencing platform, the bwa-GATK pipeline were shown to call more than twice as many variants (*n* = 52,117) as the Torrent Variant Caller (TVC) pipeline (*n* = 19,847). Variant concordance between the two pipelines was only 29.8 %. We also noted that the concordance (1.1 %) of novel variants was much lower than that of known variants (71.2 %). Although the concordance of SNPs between the two pipelines was 71.6 % among all 22,496 SNPs, the InDel concordance was extremely low, at just 1.3 % for all 32,925 InDels (Additional file 4: Table S2).

For the HiSeq 2000 sequencing platform, we also investigated the differences between variants detected by bwa-GATK and stampy-GATK pipelines. A 90.5 % concordance rate for all 24,407 variants was observed, with the overall concordance of novel variants (44.1 %) shown to be much lower than that of known variants (92.4 %) (Additional file 5: Table S3). The 92.8 % concordance of SNPs was also higher than the 57.7 % concordance of InDels.

Validated rates of three calling pipelines which differ only in read mapping, were also compared: bwa-se refers to bwa mapping of HiSeq 2000 reads to the human reference with the single-end reads mode, bwa-pe uses the paired-end read mode, and stampy-se uses stampy-1.0.22 software (http://www.well.ox.ac.uk/project-stampy) with the single-end read mode [26]. In the sets of Sanger sequencing validated variants, the bwa-pe pipeline called 130 SNPs, which was more than bwa-se and stampy-se, which called 127 and 114 SNPs, respectively. Additionally, the validated rate of SNPs called by the bwa-pe pipeline (90.0 %) was higher than that of bwa-se (86.6 %) and stampy-se (88.6 %) (Table 5).

**Table 5:** Comparison of the validation rates of variants called by different pipelines for SureSelect-HiSeq data

	bwa_pe^a^ variants		bwa_se^b^ variants		stampy_se^c^ variants	
	InDels	SNPs	InDels	SNPs	InDels	SNPs
Validated true	93.1 %(108)	90.0 %(117)	93.1 %(108)	86.6 %(110)	92.5 %(99)	88.6 %(101)
Validated false	6.9 %(8)	10.0 %(13)	6.9 %(8)	13.4 %(17)	7.5 %(8)	11.4 %(13)

Note: ^a^bwa-pe, bwa mapping with paired-end reads mode

^b^bwa-se, bwa mapping with single-end reads mode

^c^stampy-se, stampy-1.0.22 software mapping with single-end reads mode

## Discussion

Following important advances in NGS technologies and target DNA enrichment techniques [27, 28], WES is being used to identify variants associated with disease [15, 29–34]. However, few studies have comprehensively investigated the accuracy of variant calling across different sequencing platforms. This report focused on the variants detected by Proton and HiSeq 2000 combined with different exome enrichment kits.

Because of differences in the target regions between the Ion TargetSeq™ Exome Enrichment Kit and SureSelect Human All Exon V4 Kit, we considered only 33.6 Mb of overlapping regions between the two kits and evaluated the accuracy of three kinds of variant in four samples: concordant, TargetSeq-Proton-specific and SureSelect-HiSeq-specific SNVs. The results showed that ~70 % of variants were concordant and ~30 % were platform-specific. Additionally, the validation rate of concordant SNPs and 1-bp InDels was found to be highest, up to 91.5 % and 100.0 %, respectively, followed by SureSelect-HiSeq-specific (88.3 % for SNPs and 89.6 % for 1-bp InDels), then TargetSeq-Proton-specific at just 60.0 % for SNPs and 15.8 % for 1-bp InDels (Table 4).

Parla et al. previously observed that the SeqCap EZ Human Exome Kit (Roche NimbleGen) and SureSelect Human All Exon Kit performed similarly in target capturing and sequencing [35], whereas Chilamakuri et al. reported that four exonic DNA enrichment kits (NimbleGen SeqCap EZ, Agilent SureSelect, Illumina TruSeq Exome, and Nextera Exome) performed well in a systematic comparison of exome database coverage, target capturing efficiency, and GC bias [36].

In the present report, the Agilent SureSelect V4 Kit used 120-bp RNA probes with a GC content of 49.3 ± 11.1 %, whereas the Ion TargetSeq™ Exome Enrichment Kit used variable length DNA probes of 85.1 ± 64.1 bp with a GC content of 48.9 ± 12.2 %. These differences may affect the GC content of reads (Additional file 6: Table S4) and the coverage of specific loci, which can influence variant calling, although the global coverage was similar at the level of 10× sequencing depth (Table 1, Additional file 1: Figure S1). The notable discrepancy of variant calling between SureSelect-HiSeq and TargetSeq-Proton sequencing platforms can be explained in part by different capturing efficiency of exome enrichment kits and the inadequate sequencing depth of platform-specific loci. For example, of 5689 SureSelect-HiSeq-specific variants in sample S3, only 5.3 % (301) were not covered and 46.6 % (2650) were covered at ≤10× by TargetSeq-Proton reads; among 1518 TargetSeq-Proton-specific variants, 2.1 % (32) were not sequenced and 30.2 % (459) were sequenced at ≤10× by the SureSelect-HiSeq strategy. Thus, partial one platform-specific variants can also be detected by another platform when sequencing coverage increases.

The discrepancy mainly results from other factors such as characters intrinsic in sequencing platforms, read alignment and variant calling methods. Although the detailed InDel error rate was unavailable in our study, the Proton sequencing platform biases InDel errors because its underlying sequencing principle is the same as that of Ion Torrent Personal Genome Machine (PGM). In untrimmed bases of PGM, the error rate varies from 0.84 to 1.76 % for insertion errors and from 0.80 to 1.07 % for deletion errors [37]. To minimise the impact of InDel errors produced by the Proton sequencing platform, base calling using Torrent Suite Software was performed with fairly stringent filters. This decreased the number of variants detected by Proton reads, as shown by the fact that several SureSelect-HiSeq-specific validated variants were not detected by TargetSeq-Proton although they were covered by Proton reads. Compared with variants called by Torrent Suite Software, about 90 % of variants (35,574) called only by the bwa-GATK pipeline were novel small InDels, which represents a high possibility of false positives (Additional file 4: Table S2). This shows that the TVC pipeline, optimised to deal with varied length reads and error profiles specific to Proton system, processed the Proton data much better than the bwa-GATK pipeline.

Characterization of the flanking 10-bp reference regions of the 1-bp small InDels showed that ~70 % loci were in homopolymer regions (Additional file 7: Table S5). Moreover, HiSeq 2000 detected 1-bp InDels more sensitively than Proton (Additional file 3: Figure S2), even in the region with a homopolymer size of ≥10 bp. By contrast, the homopolymer size of InDel regions detected by Proton rarely exceeded 5. Our observation that Proton reads were slightly biased to InDel errors occurring in homopolymer types A and T (Additional file 8: Figure S3) was less than that previously shown for HiSeq reads [38]. This suggests that a more accurate variant calling method should be developed for use of the Proton platform to detect small InDels.

As a biologically relevant and prevalent form of genetic variation [39], more than 800,000 InDels in a diverse population have been mapped to known genes, some of which can be associated with genetic diseases [40, 41]. Our analysis revealed a substantial difference in the InDels detection ability between Proton and HiSeq 2000, which was also observed in previous studies [23]. Similarly, low concordant InDels called by different pipelines have also been reported previously [42]. The low validation rate of variants specific to TargetSeq-Proton showed that Proton has a high false positive rate of calling small InDels or SNPs. Recently, a new open source algorithm, Scalpel, has been developed [43]. This combines mapping, assembly, and repeat analysis, and is coupled with a self-tuning k-mer strategy for the sensitive and specific discovery of InDels in exome capture data. Scalpel outperforms other InDel calling approaches (such as GATK HaplotypeCaller and SOAPindel [44]) for InDel discovery, particularly in regions containing near-perfect repeats, and has the power to detect long (≥30 bp) transmitted events as well as enriching likely gene-disrupting InDels in autistic children. However, it is unknown whether Scalpel is suitable for Proton fragment reads because it was developed for Illumina HiSeq 2000 paired-end reads.

Our comparison of different SNV calling pipelines for HiSeq 2000 data revealed that two single-end mapping methods for HiSeq 2000 reads slightly decreased the number and accuracy of SNPs (Table 5). This suggests that paired-end sequencing and mapping should be performed if possible. Our data also demonstrated that HiSeq 2000 and Proton platforms are partially complementary for variant detection. To obtain truly comprehensive exonic variants, WES should be performed on different platforms with deep paired-end coverage.

## Conclusions

We detected SNPs and small InDels of four whole exomes using Torrent Suite Software 3.6.2 for TargetSeq-Proton data and using bwa-GATK for SureSelect-HiSeq data. We observed a notable discrepancy in variant calling between HiSeq 2000 and Proton sequencing platforms. A more comprehensive set of variants could be obtained by combining deep sequencing from HiSeq 2000 and Proton. Among the different subsets of variants, the Sanger sequencing validation of concordant variants was higher than that of variants specific to SureSelect-HiSeq or TargetSeq-Proton sequencing strategies. For sequencing platform-specific variants, SureSelect-HiSeq achieved a higher level of accuracy in variant calling than TargetSeq-Proton, specifically for InDel detection. The combination of deep paired-end sequencing on different sequencing platforms, alongside the development and application of multiple variant calling tools, will effectively maximise the sensitivity and specificity of variant detection in biomedical applications.

## Methods

### Sample collection and genomic DNA preparation

This study was approved by Beijing Institute of Genomics Institutional Review Board for Human Investigation under the HHS Federal Wide Assurance of Compliance Number 00014534 and IRB registration number IORG0005863. Written informed consent for participation was obtained from the participants (>18 years age) prior to sample collection.

Blood samples were collected from four individuals and genomic DNA was extracted using alkaline lysis and ethanol precipitation with the QIAamp DNA Blood Kits (Qiagen, Valencia, CA). The pure high molecular weight genomic DNA samples were quality-checked on agarose gels and quantified using a micro-volume spectrophotometer (NanoDrop 1000; Thermo Fisher Scientific Inc., West Palm Beach, FL).

### Ion TargetSeq exome enrichment and Proton Sequencing

For each sample, 3 μg high-quality genomic DNA was used to prepare the Ion TargetSeq-Exome 50 Mb capture library. Randomly fragmented genomic DNA underwent adapters-ligation, nick-repairing, and purification prior to size selection according to the manufacturer’s instructions (Ion TargetSeq Guide; Life Technologies, Carlsbad, CA). Size selection was conducted using the iBase unit Power System and the E-Gel SizeSelect 2 % Agarose Gel (Life Technologies). Library DNA was obtained and amplified according to the Ion TargetSeq Guide. The amplified product was cleaned with the Agencourt AMPure XP reagent (Beckman Coulter, Brea, CA) and quantitated and qualitatively assessed on the Agilent Bioanalyzer 2100.

A total of 500 ng of each size-selected fragment library was hybridized with pooled solution-phase DNA probes from the Ion TargetSeq™ Exome Enrichment Kit for 72 h, then the DNA was recovered, amplified, and purified according to the manufacturer’s instructions.

The enriched DNA was sequenced by the Ion Proton sequencer according to the manufacturer’s protocols. Sequencing templates were prepared on Ion OneTouch 2 and Ion OneTouch ES stations, then loaded onto the Proton PI Chip prior to sequencing.

### SureSelect Human All Exon v4 exome enrichment and HiSeq 2000 sequencing

A total of 1.5 μg of high-quality genomic DNA per sample was used in the Agilent SureSelect Human All Exon v4 kit capture process. Randomly fragmented DNA was end-repaired, extended with an ‘A’ nucleotide at the 3’end, ligated with the indexing-specific paired-end adapter and amplified according to the manufacturer’s protocol (SureSelect Target Enrichment for Illumina Multiplexed Sequencing version 1.5; Agilent Technologies, Los Angeles, CA). Exome-containing adapter-ligated libraries were hybridized with RNA baits for 24 h at 65 °C, and enriched with streptavidin-conjugated magnetic beads (Dynabeads MyOne Streptavidin T1; Invitrogen). Captured libraries were amplified, and then purified with the Agencourt AMPure XP reagent, then analysed with the Agilent Bioanalyzer 2100 to evaluate the library quality. The qualified exome-captured libraries were sequenced using HiSeq 2000 with the TruSeq PE Cluster kit v3 and TruSeq SBS kit v3 according to the manufacturer’s protocol.

### Proton data analysing with Torrent Suite software

For each Proton run, “Ion TargetSeq” was used as the application type, human reference hg19 (UCSC version of GRCh37 reference assembly) as the reference library and “Ion-TargetSeq-Exome-50 Mb-hg19_revA.bed” as the target regions bed file. Bases were called by the Torrent Suite base calling algorithm, and aligned to human reference hg19 by the Torrent Mapping Alignment Program (TMAP v3.4.1), then alignment metrics were also produced. The above base-calling and reference-aligning were performed using the default parameters. The BAM file was subsequently used to call the corresponding variants by the Torrent Variant Caller (TVC3.6.2) plugin using a standard workflow entitled “Germ Line - High Stringency”.

### Burrows–Wheeler Aligner - Genome Analysis Toolkit variant calling for Proton reads

The GRCh37 reference assembly integrating with the 1000 Genomes Project phase I analysis (human_g1k_v37 version) was downloaded from the Genome Analysis Toolkit (GATK) Resource bundle (https://www.broadinstitute.org/gatk/download/). In the target regions of two exome-capturing kits, no differences are found between references of human_g1k_v37 and hg19. Proton reads were aligned to human_g1k_v37 using the Burrows-Wheeler Aligner (bwa) software version 0.6.2 (http://bio-bwa.sourceforge.net/) with single-end reads mode [45, 46]. Duplicate reads based on paired ends aligning to the same start locations because of either optical or PCR artefacts were marked and excluded from further analysis using the MarkDuplicates module of Picard software version 1.70 (http://broadinstitute.github.io/picard/). GATK v2.5-2 was applied to re-calibrate the base quality score, realign reads around known and novel sites of InDel polymorphisms, and perform SNP and InDel discovery and genotyping using standard hard filtering parameters according to GATK Best Practices recommendations [47–49]. GATK was used to filter high quality InDels by hard criteria: “QD < 2.0, ReadPosRankSum < -20.0 FS > 200.0” and SNPs by hard criteria: “QD < 2.0, MQ < 40.0, FS > 60.0, HaplotypeScore > 13.0, MQRankSum < -12.5, ReadPosRankSum < -8.0”.

### Variant calling pipelines for HiSeq 2000 reads

When HiSeq 2000 and Proton platforms were compared, HiSeq 2000 reads were aligned to human_g1k_v37 using bwa v0.6.2 with the paired-end reads mode. While comparing different pipelines calling variants for HiSeq 2000, reads were aligned to human_g1k_v37 using bwa v0.6.2 with the single-end reads mode. BAM sorting, duplicate read marking, realignment, base quality recalibration, variant calling and filtering were performed with the same parameters used for the bwa-GATK variant calling pipeline in Proton reads.

### Validation of variants by Sanger sequencing

From the four samples, 240 SNPs and 240 small InDels were picked for variant validation by Sanger sequencing. Amplicons of ~200 bp containing the variants were designed using AssayDesigner software (SEQUENOM Inc., USA) with default parameters. PCR products were sequenced by Sanger chemistry using the 3730XL sequencer. All variants were manually called. Heterozygous InDels produce a complex signal on the chromatogram displaying multiple heterozygous peaks similar to substitution polymorphisms and a secondary peak corresponding to the base in the alternate allele [50]. This complex signal was applied to recognize heterozygous InDels.

Accession No. All exome sequencing data in this report are available at the NCBI under accession SRP052890.

## Additional files

Additional file 1: Figure S1. The cumulative coverage proportion of target regions in 4 samples. The proportion of target region for specific cumulative coverage, was caculated and were showed at the coverage from 0× to 200× in for sample S1 (A), sample S2 (B), sample S3 (C) and sample S4 (D). (PNG 196 kb) Additional file 2: Table S1. Ti/Tv ratios statistics for SNPs. (XLSX 9 kb) Additional file 3: Figure S2. The occurence of homopolymer size of 1-bp InDels detected by TargetSeq-Proton and SureSelect-HiSeq in sample 3. The length and kinds (poly-A, poly-T, poly-G and poly-C) of the homopolymer, matching the genomic location of and identical to the base of 1 bp insertions or deletions, was caculated. The homopolymers with different size in the 33.6 Mb overlapping exonic regions were counted for sample S3. (PNG 72 kb) Additional file 4: Table S2. Comparison of calling variants between TVC and bwa_GATK tools for reads produced by Proton. (XLSX 9 kb) Additional file 5: Table S3. Comparison of concordant variants between softwares for reads produced by HiSeq 2000. (XLSX 9 kb) Additional file 6: Table S4. The distribution of GC content and length for reads produced by Proton and HiSeq 2000. (XLSX 8 kb) Additional file 7: Table S5. The statistics for detected 1-bp InDels in homopolymer and non-homopolymer regions. (XLSX 8 kb) Additional file 8: Figure S3. Percentage of reads containing InDels in regions of homopolymers (homopolymer size ≥5) in TargetSeq-Proton and SureSelect-HiSeq data in sample S3. (PNG 19 kb)
