# Supplementary figures and images for: Comparison and evaluation of two exome capture kits and sequencing platforms for variant calling
Source: BMC Genomics. 2015 Aug 5;16(1):581. doi: 10.1186/s12864-015-1796-6 (PMC4524363; doi:10.1186/s12864-015-1796-6)

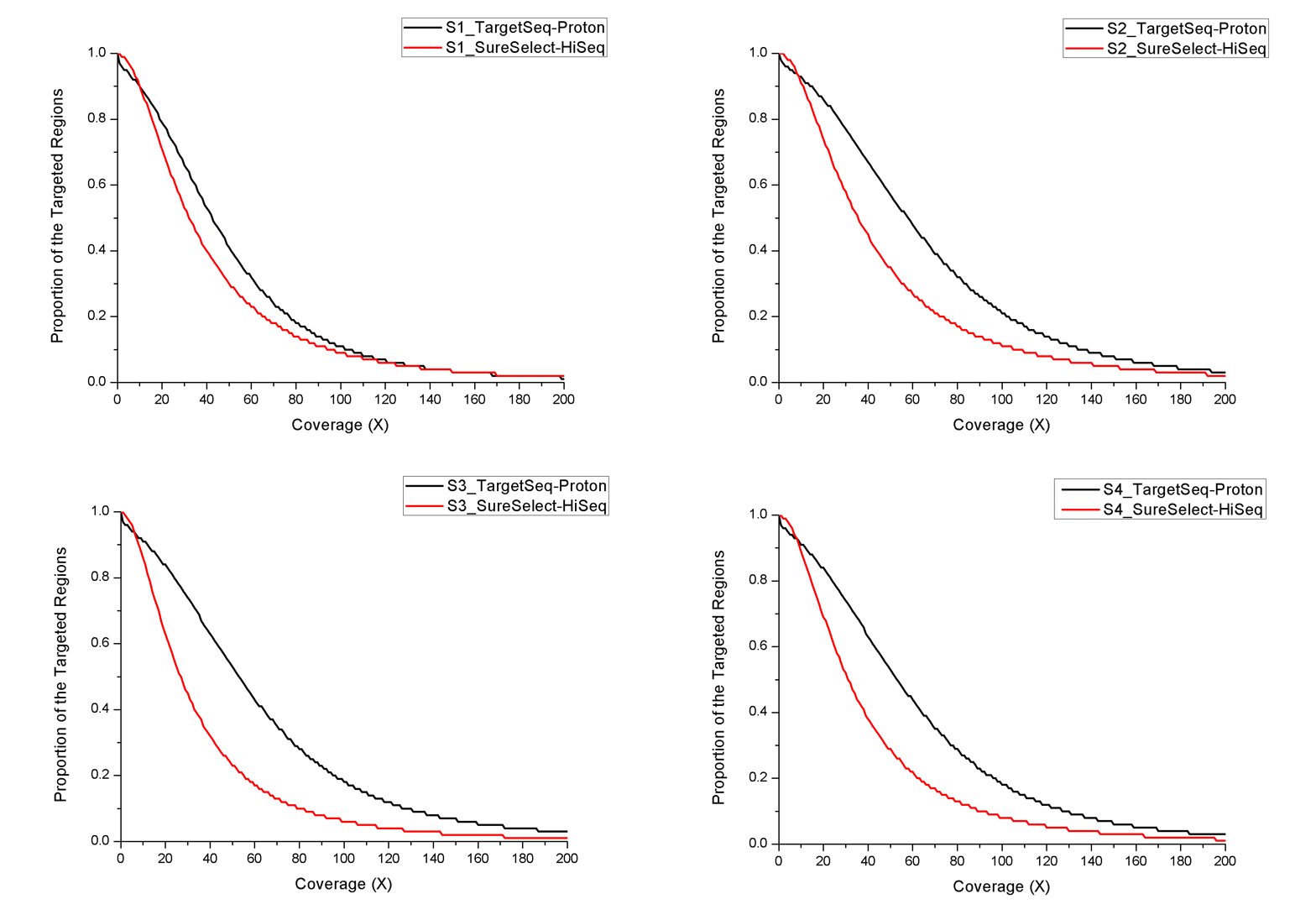

Supplement: Additional file 1: Figure S1. — The cumulative coverage proportion of target regions in 4 samples. The proportion of target region for specific cumulative coverage, was caculated and were showed at the coverage from 0× to 200× in for sample S1 (A), sample S2 (B), sample S3 (C) and sample S4 (D). (PNG 196 kb) [file 12864_2015_1796_MOESM1_ESM.png]

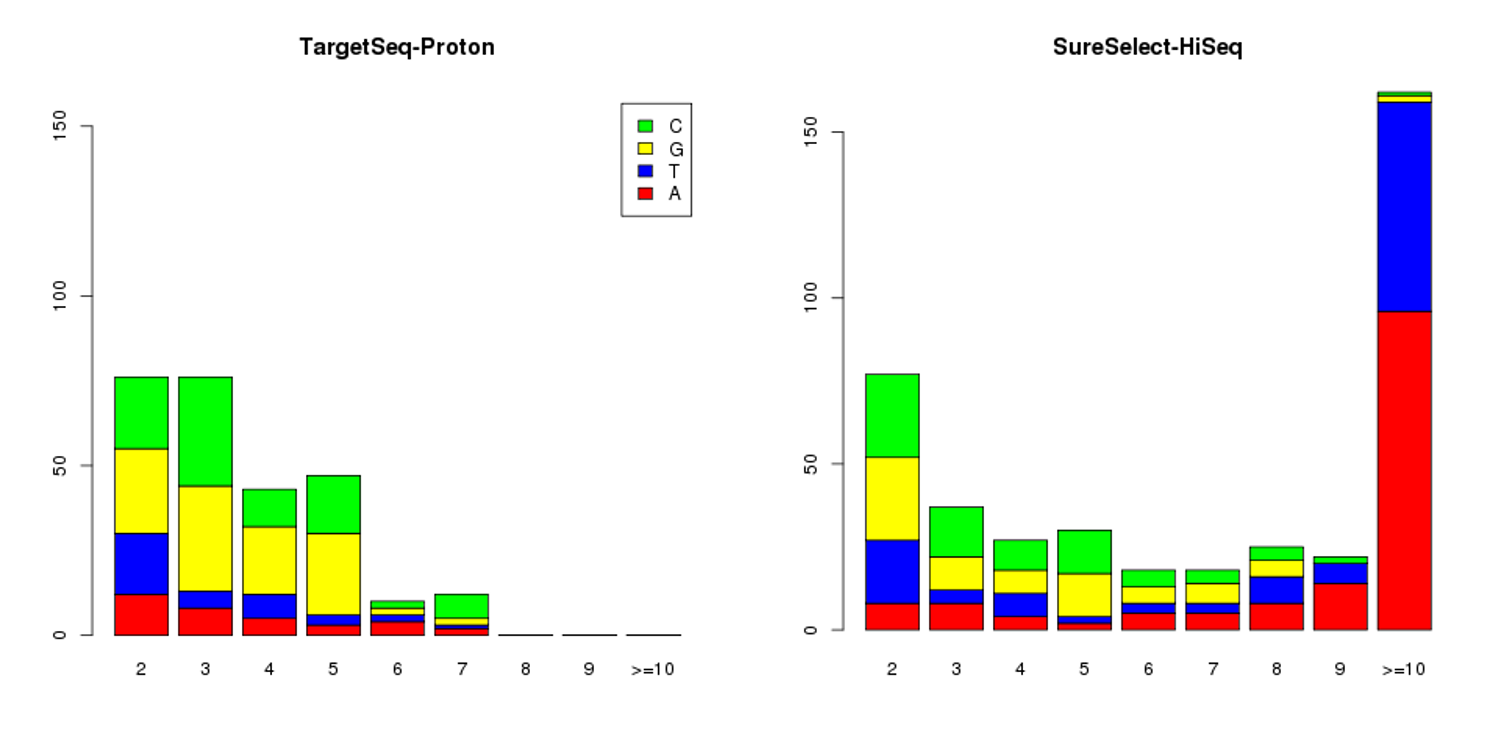

Supplement: Additional file 3: Figure S2. — The occurence of homopolymer size of 1-bp InDels detected by TargetSeq-Proton and SureSelect-HiSeq in sample 3. The length and kinds (poly-A, poly-T, poly-G and poly-C) of the homopolymer, matching the genomic location of and identical to the base of 1 bp insertions or deletions, was caculated. The homopolymers with different size in the 33.6 Mb overlapping exonic regions were counted for sample S3. (PNG 72 kb) [file 12864_2015_1796_MOESM3_ESM.png]

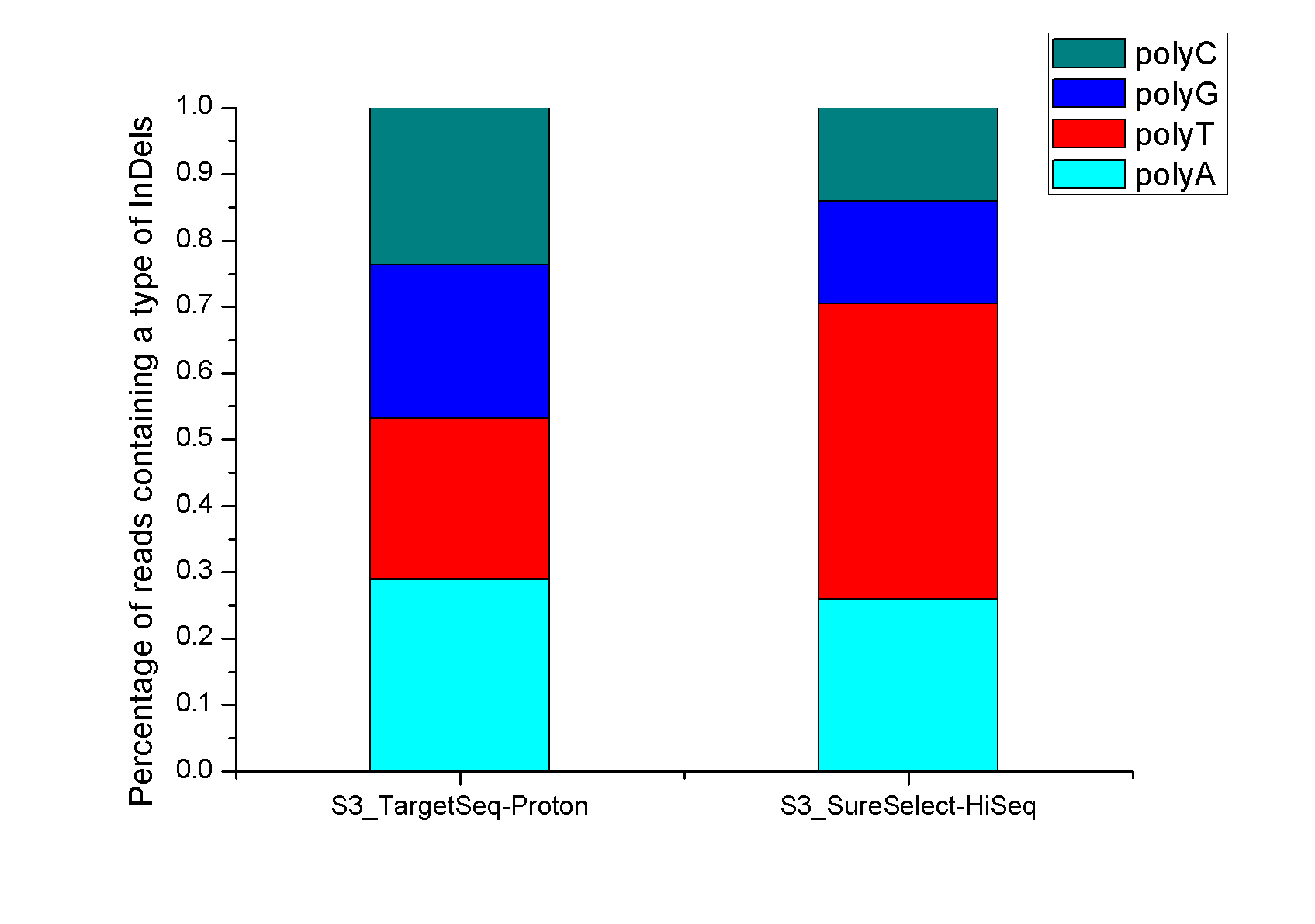

Supplement: Additional file 8: Figure S3. — Percentage of reads containing InDels in regions of homopolymers (homopolymer size ≥5) in TargetSeq-Proton and SureSelect-HiSeq data in sample S3. (PNG 19 kb) [file 12864_2015_1796_MOESM8_ESM.png]
